# Western Iowa Veterinary Medical Association

**Published:** 1891-09

**Authors:** G. A. Johnson

**Affiliations:** Acting-Secretary; Carroll, Ia.


*Western Iowa Veterinary Medical Association.*—Pursuant of a scheme to organize a veterinary association in the western part of Iowa, Dr. S. H. Johnston, of Carroll, issued a call for a meeting of the graduated veterinarians of this part of the State, to meet at that place on the 24th of June, 1891.

Notwithstanding that several had promised to attend, when the time appointed arrived, there were but two present, Drs. S. H. Johnston, of Carroll; and G. A. Johnson, of Odebolt.

Not wishing to let the matter drop, they drafted a constitution and by-laws, and appointed the following officers: President, Dr. S. H. Johnston; Vice-President, Dr. Gibson; Secretary and Treasurer, Dr. G. A. Johnson; and set August 8th, 1891, as a date for a second meeting, at which time the proceedings of the June meeting would be presented for adoption.

G. A. JOHNSON, *Acting-Secretary.*

---

CARROLL, Ia., Aug. 8, 1891.

Second meeting of the Western Iowa Veterinary Medical Association. Meeting called to order by Acting-President Johnston.

After some slight changes in the constitution and by-laws, the proceedings of the June meeting were adopted.

Moved by Dr. Johnson and seconded by Dr. Gibson, that the president procure a list of service-fees from the Ontario Veterinay College, and one from the Iowa State M.D. Association, carried.

After some further routine business, Dr. G. A. Johnson, read a paper on "Open-Joints," and a "New Remedy," which elicited a lively discussion, after which the meeting adjourned to meet again at the call of the secretary.

This will be but a small association of a dozen or so of members, as that is about the number of graduated veterinarians in this section of the State. The following are the members at present: S. H. Johnston, of Carroll, President; J. I. Gibson, of Denison, vice-President; G. A. Johnson, of Odebolt, Secretary-Treasurer. Also L. A. Thomas, of Atlantic; and S. S. Stewart, of Council Bluffs.

There will probably be several more applicants at the next meeting.

G. A. JOHNSON, *Secretary*

